# Prenatal activation of Toll-like receptors-3 by administration of the viral mimetic poly(I:C) changes synaptic proteins, N-methyl-D-aspartate receptors and neurogenesis markers in offspring

**DOI:** 10.1186/1756-6606-5-22

**Published:** 2012-06-09

**Authors:** Caroline M Forrest, Omari S Khalil, Mazura Pisar, Robert A Smith, Lynda Gail Darlington, Trevor W Stone

**Affiliations:** 1 Institute for Neuroscience and Psychology, University of Glasgow, West Medical Building, Glasgow, G12 8QQ, UK; 2 Epsom General Hospital, Epsom, KT18 7EG, UK

**Keywords:** Poly(I:C), Development, Synaptophysin, Synaptotagmin, Rho proteins, Eph receptors

## Abstract

**Background:**

There is mounting evidence for a neurodevelopmental basis for disorders such as autism and schizophrenia, in which prenatal or early postnatal events may influence brain development and predispose the young to develop these and related disorders. We have now investigated the effect of a prenatal immune challenge on brain development in the offspring. Pregnant rats were treated with the double-stranded RNA polyinosinic:polycytidylic acid (poly(I:C); 10 mg/kg) which mimics immune activation occurring after activation of Toll-like receptors-3 (TLR3) by viral infection. Injections were made in late gestation (embryonic days E14, E16 and E18), after which parturition proceeded naturally and the young were allowed to develop up to the time of weaning at postnatal day 21 (P21). The brains of these animals were then removed to assess the expression of 13 different neurodevelopmental molecules by immunoblotting.

**Results:**

Measurement of cytokine levels in the maternal blood 5 hours after an injection of poly(I:C) showed significantly increased levels of monocyte chemoattractant protein-1 (MCP-1), confirming immune activation. In the P21 offspring, significant changes were detected in the expression of GluN1 subunits of NMDA receptors, with no difference in GluN2A or GluN2B subunits or the postsynaptic density protein PSD-95 and no change in the levels of the related small GTPases RhoA or RhoB, or the NMDA receptor modulator EphA4. Among presynaptic molecules, a significant increase in Vesicle Associated Membrane Protein-1 (VAMP-1; synaptobrevin) was seen, with no change in synaptophysin or synaptotagmin. Proliferating Cell Nuclear Antigen (PCNA), as well as the neurogenesis marker doublecortin were unchanged, although Sox-2 levels were increased, suggesting possible changes in the rate of new cell differentiation.

**Conclusions:**

The results reveal the induction by prenatal poly(I:C) of selective molecular changes in the brains of P21 offspring, affecting primarily molecules associated with neuronal development and synaptic transmission. These changes may contribute to the behavioural abnormalities that have been reported in adult animals after exposure to poly(I:C) and which resemble symptoms seen in schizophrenia and related disorders.

## Background

A substantial amount of clinical epidemiological evidence has suggested that human maternal infection during pregnancy can adversely affect brain development of the offspring in postnatal life [1,2]. Maternal infection could, therefore, represent an important contributor to the emergence of neurodevelopmental disorders including schizophrenia, autism spectrum disorders and depression [3-5]. This hypothesis is supported by experimental evidence from rodent models that infection during gestation may lead to behavioural changes in the offspring which may not be apparent until adulthood. Those behavioural abnormalities often resemble features of the neurodevelopmental disorders seen in humans [6-8]. In order to prevent or treat neurodevelopmental disorders of this kind it will be essential to understand the molecular changes by which infection of the mother, foetus or neonate influences brain development and its subsequent maturation.

Animal models for the effects of infection on development have been described [9,10]. Many infections of pregnancy are caused by viruses, the effects of which can be simulated by the synthetic viral coat-mimetic poly-[inosinic acid:cytidylic acid], (poly(I:C)). This double-stranded RNA molecule activates Toll-Like Receptor-3 (TLR-3) on dendritic cells, macrophages and B cells, leading to activation of the immune system. The effects of poly(I:C) include inducing the expression of interferon-γ (IFN-γ) and other major inflammatory mediators such as interleukin-1β (IL-1β), IL-6 and tumour necrosis factor-α (TNF-α), as well as leucocyte chemoattractants such as monocyte chemotactic protein-1 (MCP-1) [11], thus reproducing many features of a full infective immune response.

In the present study pregnant female rats were treated with poly(I:C) during the late phases of gestation. Parturition was then allowed to proceed normally and the offspring were allowed to grow until weaning (postnatal day 21; P21) when the expression of molecules known to play critical roles in early brain development or which reflect the formation of synaptic contacts were studied. The results indicate that several of these molecules show substantial changes which persist at least until the time of weaning and which may underlie aspects of the behavioural abnormalities reported previously.

## Methods

### Animals

This study was carried out according to the regulations of the Animals (Scientific Procedures) act 1986 of the UK, administered and monitored by the Home Office. The study was performed according to internationally recognized procedures and was approved by the University of Glasgow Research Ethics Committee. Male and female Wistar rats were housed together for mating and inspected daily for the occurrence of a vaginal plug. The pregnant females were housed alone from that point, with free access to food and water. Preliminary experiments established that 10 mg/kg of poly(I:C) could be given to pregnant dams during the last seven days of gestation with no signs of stress, behavioural changes or abnormal behaviour towards the neonatal pups after birth.

A dose of 10 mg/kg was used partly based on the existing literature reporting that doses of 5 or 10 mg/kg are able to induce a clear inflammatory response in rodents [12] whereas lower doses are generally ineffective. However, much of this previous work was performed in normal adult or neonatal animals, and involved single injections whereas we wished to develop a model in which the immune system was activated over a time period of several days. We therefore performed preliminary experiments using different doses and injection schedules of poly(I:C) specifically in pregnant rats during late gestation. The results supported the adult data, firstly confirming that 10 mg/kg could be used without evoking any sickness behaviour (hyperpyrexia, lethargy, hyper-sensitivity, anorexia) after a single injection and showing that the same dose could be administered on three separate occasions with a similar lack of adverse effects on the pregnant dam. This provides confidence that our dose is well-tolerated, and it was therefore selected to give the maximum chance of seeing changes in the offspring. The acceptability of this dose was confirmed by the fact that litter sizes were not affected, with an average litter size of 10.4 in saline-treated dams compared with 10.33 in animals receiving poly(I:C), confirming that the 10 mg/kg dose was having no adverse effects on mother or litter in utero or on the progress of gestation.

Poly(I:C; P0913) was obtained from Sigma (Poole, UK) and was prepared for injection by dissolving 10 mg in 1 ml of sterile saline (0.9% w/v NaCl). It was heated to 50°C to ensure complete solubility and allowed to cool to room temperature to allow re-annealing of the double-stranded RNA. Pregnant rats were injected intraperitoneally with 10 mg/kg poly(I:C) or vehicle (sterile saline 0.9%) on gestation days E14, E16 and E18 in order to extend the temporal impact of maternal immune activation on the embryos, with no apparent ill effects on the dam or her behaviour towards her litter after birth. All injections were administered between 9 am and 11 am to minimise variation in responses. Following the injections, gestation was allowed to proceed normally, with neonates being allowed to survive until weaning (postnatal day 21; P21) when they were taken from the home cage for euthanasia followed by removal of the brain. Each brain was divided into the two cerebral hemispheres and frozen immediately on dry ice before being transferred to storage at −80°C until required for analysis. Each member of a litter was treated identically, with whole litters being taken at the same time so that no animal would experience the possible trauma of losing littermates while themselves surviving to a later date. This protocol also ensured that changes of maternal behaviour caused by the removal of some pups could not affect the development of survivors.

### Cytokine analysis

For assessment of cytokine production following poly(I:C) challenge, pregnant females were administered a single injection of either poly(I:C) 10 mg/kg or vehicle (sterile saline 0.9%) on gestational day E18. Rats were sacrificed 5 h later and blood was collected by cardiac puncture using sterile syringes into sterile tubes containing 20U/ml heparin. The samples were then centrifuged at 600 *g* for 15 minutes, and maternal plasma was stored at −80°C until required for cytokine analysis.

The levels of the pro-inflammatory cytokines interleukin-1β (IL-1β) and tumour necrosis factor-α (TNF-α), and the chemokine monocyte chemoattractant protein-1 (MCP-1) were analysed using Signosis ELISA profiling kits (Caltag Medsystems, Buckingham, UK). The relative amounts of these proteins present in the maternal plasma of pregnant animals treated with vehicle or poly(I:C) were calculated from the optical densities read at 450 nm using a microplate reader.

### Immunoblotting

Brain sample homogenates were prepared in RIPA buffer (50 mM Tris, 150 mM NaCl, 0.1% SDS, 0.5% Triton X-100, 1% IGEPAL, and a Roche complete protease inhibitor tablet) and centrifuged at 18000 *g* for 5 min at 4°C. Supernatants were collected for protein concentration determination using the Bio-Rad Coomassie Blue protein assay (Bio-Rad, Hemel Hempstead, UK). Samples were then normalised to 10 μg and prepared as; 65% protein sample, 25% sample buffer and 10% reducing agent (Life Technologies, Paisley, UK), and heated at 70°C for 10 min. The protein samples were loaded onto NuPAGE Novex 4–12% Bis-Tris (1.0 mm) 15 lane gels (Life Technologies, Paisley, UK) and run at 150 volts for 80 min to separate proteins according to their molecular weight. SeeBlue pre-stained standard (Life Technologies, Paisley, UK) was included on each gel as a molecular weight marker. The separated proteins were then blotted onto Invitrolon poly(vinylidene difluoride) membranes (Life Technologies, Paisley, UK) at 30 V for 60 min. The membranes were blocked for 1 h in 5% non-fat dried milk solution in Tris-buffered saline containing 0.05% Tween (TBST), before overnight incubation at 4°C with the appropriate primary antibody (diluted in 5% milk-TBST). Membranes were then washed 3 times for 15 min with TBST and incubated with the appropriate horseradish peroxidase (HRP) conjugated secondary antibody (prepared in 5% milk-TBST) for 1 h at room temperature. Following secondary antibody incubation, blots were washed 3 times for 15 min with TBST then visualised using Enhanced Chemiluminescence Plus detection kit (GE Healthcare, Chalfont St Giles, UK).

### Antibodies

Western blot analysis was carried out using the following primary antibodies raised against target proteins: GluN1 (mouse monoclonal, 05–432, 1 : 1000 dilution) and synaptophysin (mouse monoclonal, MAB368, 1 : 40000 dilution) (Millipore, Watford, UK); GluN2A (rabbit polyclonal, PPS012, 1 : 5000 dilution), GluN2B (rabbit polyclonal, PPS013, 1 : 5000 dilution), VAMP-1/synaptobrevin (goat polyclonal, AF4828, 1 : 10000 dilution), and synaptotagmin (mouse monoclonal, MAB43641, 1 : 5000 dilution) (R&D Systems, Abingdon, UK); PSD-95 (rabbit monoclonal, 3450, 1 : 10000 dilution) (Cell Signalling, New England Biolabs, Hitchin, UK); RhoA (mouse monoclonal, sc-418, 1 : 1000 dilution), RhoB (mouse monoclonal, sc-8048, 1 : 1000 dilution), EphA4 (rabbit polyclonal, sc-921, 1 : 5000 dilution), PCNA (mouse monoclonal, sc-56, 1 : 1000 dilution), doublecortin (goat polyclonal, sc-8066, 1 : 1000 dilution), Sox-2 (goat polyclonal, sc-17320, 1 : 500 dilution) and actin (goat polyclonal, sc-1615, 1 : 10000 dilution) (Santa Cruz, Insight Biotechnology, Wembley, UK). The following secondary HRP-conjugated antibodies were used at 1: 5000 dilution: goat anti-rabbit HRP (12–348) (Millipore, Watford, UK); donkey anti-goat HRP (sc-2020) and goat anti-mouse (sc-2005) (Santa Cruz, Insight Biotechnology, Wembley, UK).

### Data analysis and statistics

The western blots were analysed using Image J densitometric software (http://rsb.info.nih.gov/ij/). In order to control for variations in the total amount of protein loaded onto gels, all membranes were stained with Ponceau S solution (Sigma, Poole, UK). In addition, actin expression was examined in each series of blots and all results are expressed as the ratio of the intensity of target protein relative to actin intensity. Three pregnant rats were injected in each of the treatment groups and two pups from each litter were randomly selected for western blot analysis. Statistical comparisons were made, using Instat software, between groups of pups born to mothers treated with poly(I:C) (n = 5–6) and groups born to mothers injected with saline vehicle (n = 5–6). This protocol allowed the use of an unpaired *t*-test to examine differences between the two. A probability value of approximately 0.05 was adopted as the working criterion for significance, but precise *p*-values are indicated when >0.0001.

## Results

### Cytokine levels

**Figure 1:**
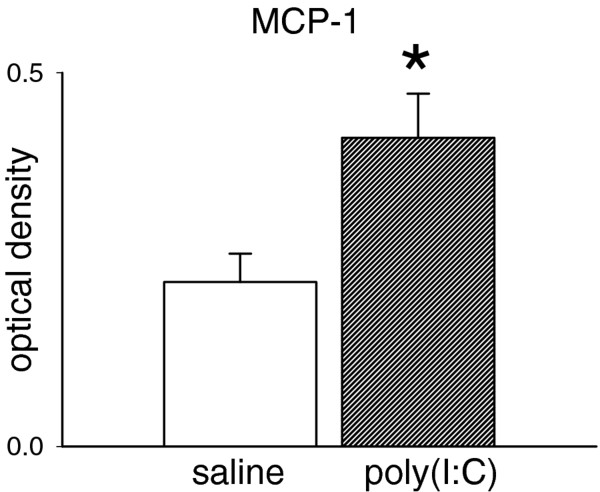
**Maternal levels of MCP-1.** Bar chart showing the maternal blood levels of monocyte chemoattractant protein-1 (MCP-1) measured 5 h following the administration of poly(I:C) 10 mg/kg. Values are shown as the mean ± s.e.mean (n = 3) in arbitrary units of optical density (OD).

The time point chosen for confirmation of a systemic inflammatory response to poly(I:C) – 5 hours following the RNA injection – was intended to highlight changes in one of the most important and well-recognised chemokines, monocyte chemoattractant protein-1 (MCP-1). The levels of this compound in the maternal blood is summarised in Figure 1, which indicates a significant increase in concentration, confirming the efficacy of the poly(I:C) dose used here. At this 5 h time point, no changes were noted in the levels of interleukin-1β (IL-1β) or TNF-α. This is consistent with previous work showing that an increase in TNF-α reaches a peak around 2-3 h following poly(I:C) administration in vivo [12,13], while others have reported no change in IL-1β levels after injection of poly(I:C) [14,15]. 

The fact that levels of IL-1β and TNF-α were not increased does confirm that the animals used in this study were not experiencing any ongoing, baseline inflammatory activity – if there were any endogenous infection or inflammatory condition such as arthritis, there would be a chronic and maintained increase of these cytokines that would be detectable before and after the injection of poly(I:C). On the other hand, it should be emphasised that these comments apply to maternal blood since brains from the embryos were not examined in this study and we cannot exclude the possibility that changes occurred in the foetal brain that are not mirrored in the maternal circulation.

### Neuronal proteins

The expression of selected target molecules was examined using Western blotting. The molecules examined are major components of the synaptic vesicle and neurotransmitter release machinery such as synaptophysin and Vesicle Associated Membrane Protein-1 (VAMP-1; synaptobrevin) as well as the vesicular release calcium sensor synaptotagmin. In addition, since N-methyl-D-aspartate (NMDA) receptors are known to play important roles in neurite outgrowth and guidance as well as synapse formation, the GluN1 subunit was examined, together with the Post-Synaptic Density molecule-95 (PSD-95), a major component of the post-synaptic complex that includes and modulates NMDA receptor function. We have also sought changes in the two major small GTPases, RhoA and RhoB, both of which have been implicated in the regulation of cytoskeletal proteins and which have been shown to be involved in synaptic transmission and plasticity [16,17].

**Figure 2:**
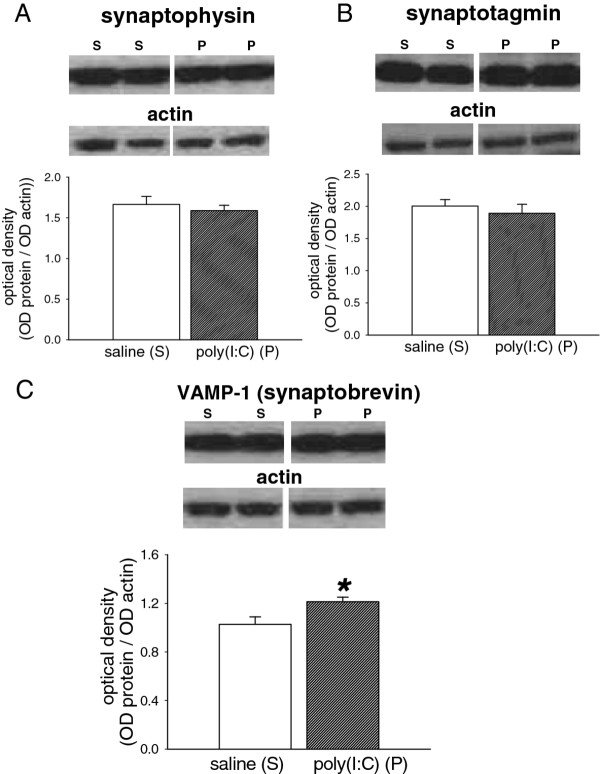
**Expression of synaptic proteins.** Bar charts showing the quantified expression of (A) synaptophysin (M.W.40 kDa) (B) synaptotagmin (M.W. 57 kDa) and (C) Vesicle Associated Membrane Protein-1 (VAMP-1; synaptobrevin; M.W. 14 kDa) in the brains of P21 rat offspring after treating the mothers with poly(I:C) 10 mg/kg on days E14, E16 and E18 of gestation. The bars indicate the mean ± s.e.mean (n = 5–6) in arbitrary units of optical density (OD) expressed as the ratio of test protein to actin. Sample western blots above each chart illustrate the data obtained from animals exposed to the saline vehicle (S) or poly(I:C) (P) and show the relevant protein and the corresponding actin blot used as a housekeeping marker * *P* < 0.05

In the brains from P21 pups exposed to poly(I:C), the expression of neither synaptophysin (*p* = 0.54; Figure 2A) nor synaptotagmin (*p* = 0.56; Figure 2B) showed any significant difference from control pups from saline-treated dams. VAMP-1, however, showed a significant increase compared with controls (*p* = 0.027; Figure 2C).

**Figure 3:**
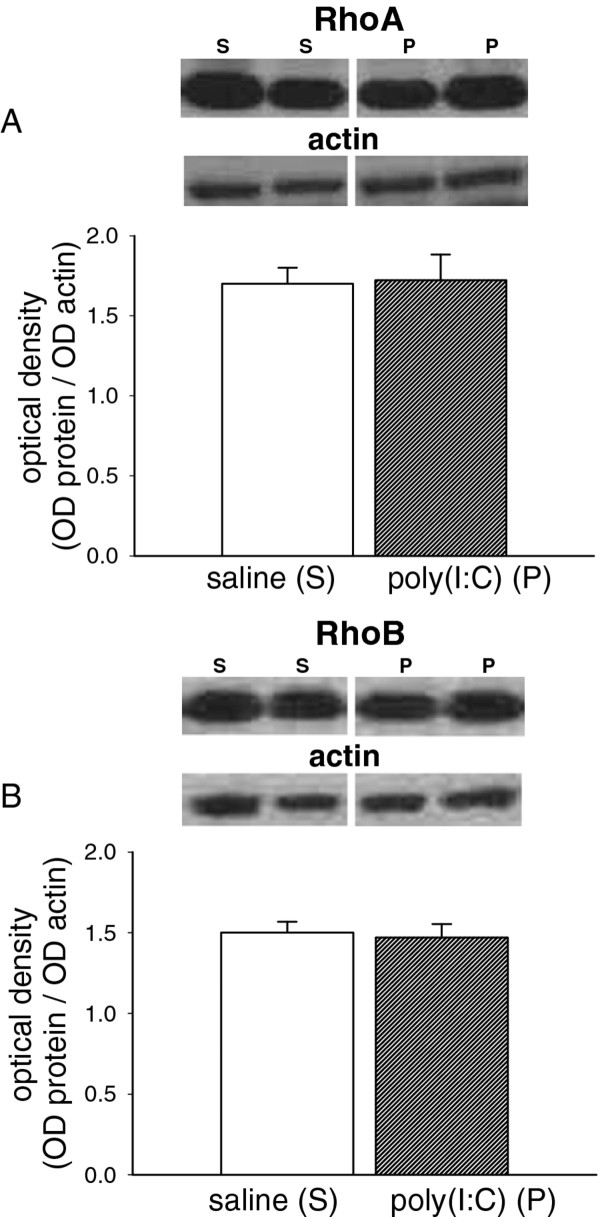
**Expression of Rho proteins.** Bar charts showing the quantified expression of (A) RhoA (M.W. 24 kDa) and (B) RhoB (M.W. 25 kDa) in the brains of P21 rat offspring after treating the mothers with poly(I:C) 10 mg/kg on days E14, E16 and E18 of gestation. The bars indicate the mean ± s.e.mean (n = 5–6) in arbitrary units of optical density (OD) expressed as the ratio of test protein to actin. Sample western blots above each chart illustrate the data obtained from animals exposed to the saline vehicle (S) or poly(I:C) (P) and show the relevant protein and the corresponding actin blot used as a housekeeping marker.

Two of the small RhoGTPase enzymes were also examined but there was no significant difference in the expression of either RhoA (*p* = 0.91, Figure 3A) or RhoB (*p* = 0.78, Figure 3B) between poly(I:C)-treated and control animals.

**Figure 4:**
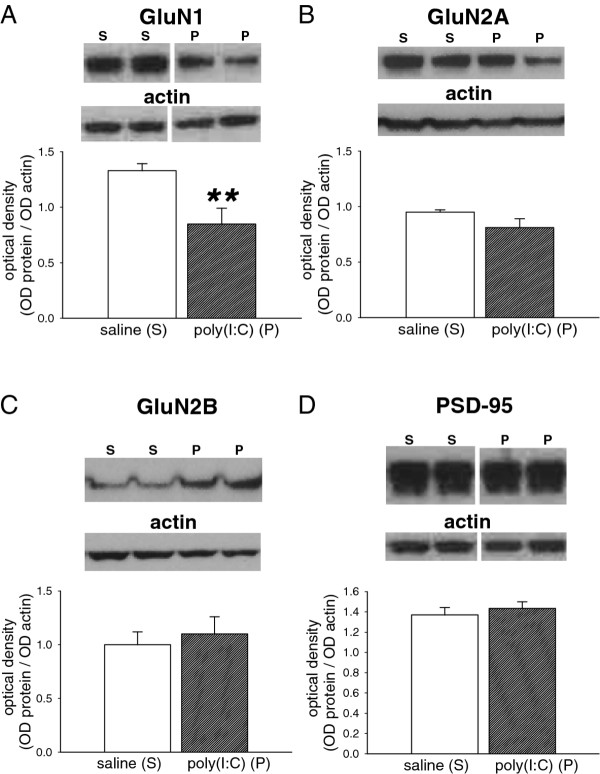
**Expression of NMDA receptor-associated proteins.** Bar charts showing the quantified expression of (A) GluN1 (M.W. 130 kDa) (B) GluN2A (M.W. 180 kDa) (C) GluN2B (M.W. 180 kDa) and (D) PSD-95 (M.W. 95 kDa) in the brains of P21 rat offspring after treating the mothers with poly(I:C) 10 mg/kg on days E14, E16 and E18 of gestation. The bars indicate the mean ± s.e.mean (n = 6, except GluN1 poly(I:C) where n = 5) in arbitrary units of optical density (OD) expressed as the ratio of test protein to actin. Sample western blots above each chart illustrate the data obtained from animals exposed to the saline vehicle (S) or poly(I:C) (P) and show the relevant protein and the corresponding actin blot used as a housekeeping marker ** *P* < 0.01.

The greatest change noted in these animals was of the GluN1 subunit of NMDA receptors, which showed a highly significant decrease compared with pups from vehicle-treated dams (*p* = 0.01; Figure 4A). In contrast, expression of the GluN2A (*p* = 0.12; Figure 4B) and GluN2B (*p* = 0.64; Figure 4C) subunits were not altered in pups exposed to poly(I:C). Similarly, expression of the post-synaptic density marker PSD-95 showed no change between controls and treated pups (*p* = 0.51; Figure 4D).

**Figure 5:**
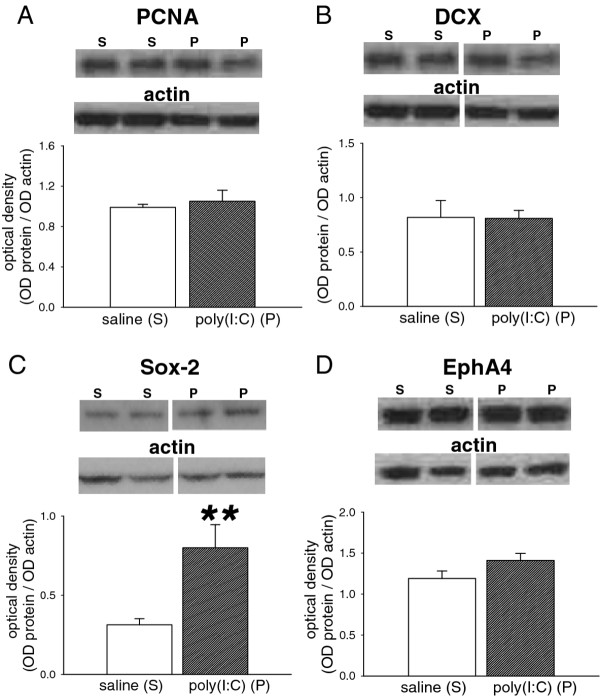
**Expression of neurodevelopmental proteins.** Bar charts showing the quantified expression of (A) PCNA (M.W. 36 kDa) (B) doublecortin (DCX; M.W. 40 kDa) (C) Sox-2 (M.W. 34 kDa) and (D) EphA4 (M.W. 120 kDa) in the brains of P21 rat offspring after treating the mothers with poly(I:C) 10 mg/kg on days E14, E16 and E18 of gestation. The bars indicate the mean ± s.e.mean (n = 6, except EphA4 poly(I:C) where n = 5) in arbitrary units of optical density (OD) expressed as the ratio of test protein to actin. Sample western blots above each chart illustrate the data obtained from animals exposed to the saline vehicle (S) or poly(I:C) (P) and show the relevant protein and the corresponding actin blot used as a housekeeping marker ** *P* < 0.01.

In addition to the above molecules, which have clearly documented roles in the early formation of synaptic contacts and in functional synaptic transmission, we have examined several molecules with more generic roles in neuronal proliferation of maturation. Proliferating Cell Nuclear Antigen (PCNA) is widely used as a marker of neurogenesis but its expression was not altered significantly following poly(I:C) (*p* = 0.64; Figure 5A). The levels of doublecortin, an intracellular protein required for neuronal migration during prenatal and early post-natal development, was not affected by poly(I:C) treatment (*p* = 0.95; Figure 5B).

Also within this group of markers is the sox-2 protein which has been implicated in neurogenesis in the brain [18] and cells deficient in sox-2 exhibit reduced neurogenesis [19]. Following exposure to poly(I:C) in utero, the expression of Sox-2 was significantly increased in the P21 pups compared with control neonates (*p* = 0.01, Figure 5C).

Finally, EphA4 expression was examined since several members of the ephrin ligands and their Eph receptors are associated with axogenesis, synapse formation and function. In pups exposed to poly(I:C) expression of EphA4 was unchanged relative to saline-treated pups (*p* = 0.12; Figure 5D).

## Discussion

The generation of a maternal immune or inflammatory response during pregnancy leads to a range of changes in the brains of the offspring which have strong parallels with those found in human behavioural disorders such as autism and schizophrenia. The administration of either bacterial lipopolysaccharides or the viral mimetic poly(I:C), (compounds frequently used in models of prenatal infection) can induce electrophysiological alterations in synaptic transmission [20-22] and elements of cognitive function [21,23,24] as well as changes in the levels of some major neurotransmitters [25]. Here we have used poly(I:C) to activate an immune response via TLR-3 in the late stages of gestation, a time when susceptibility of the rat foetal brain is believed to be comparable with that of the human brain in the second trimester of pregnancy, since brain development is at a comparable stage [26].

The measurement of cytokine and chemokine levels here confirms the ability of poly(I:C) to activate the innate immune system since the expression of MCP-1 is increased 5 h after its injection. The lack of any change in IL-1β or TNF-α levels confirms the absence of any on-going, background immune system activity, as in vivo administration of poly(I:C) is known to induce a rapid and transient increase in plasma TNF-α, peaking around 2–3 h post-injection, with levels reportedly returned to control values by 4–6 h [13-15]. In addition, Gilmore and co-workers [12] have previously shown that administration of Poly(I:C) to pregnant rats at E16 significantly elevated TNF-α in the maternal plasma at 2 h post-injection, with no increase detectable by 8 h. Poly(I:C) administered to mice at 12 mg/kg has previously been reported to induce plasma IL-1β 3 h post-treatment [13], whereas, in agreement with our finding, other groups have failed to detect increases in this cytokine after poly(I:C) administration in vivo [14,15]. Examination of mRNA for cytokines after treatment with poly(I:C) also suggests a transient profile, with peak up-regulation of genes occurring 3-6 h after poly(I:C), with only a few genes remaining elevated 24 h post-injection [27].

On the other hand, it should be emphasised that these comments apply to maternal blood since brains from the embryos were not examined in this study. Thus, we cannot exclude the possibility that changes occurred in the foetal brain that are not mirrored in the maternal circulation, although we are not aware of any previous evidence for this. However, we can exclude the initiation of an inflammatory response that persists from the administration of poly(I:C) through to the P21 stage when the offspring were examined. This is based partly on published data showing that the elevation of cytokines does not persist much beyond 24hours after injection. In addition, we have noted that there is no change of inflammatory markers such as cyclo-oxygenase-2 or NFkB at P21 as would be the case if there existed a continuing activation of the immune system (Pisar, Forrest, Omari, Darlington and Stone, unpublished observations).

It is clear from the present study that a number of significant changes are induced by the inflammatory challenge. The synaptic vesicle proteins synaptophysin, synaptotagmin and VAMP-1 (synaptobrevin), are of particular interest in brain development, and VAMP-1 has been noted as one of 18 proteins whose gene increases continually from postnatal day P0 to P45. Clear changes in several synaptic vesicle release proteins have been demonstrated in brain tissue from schizophrenic patients [28-30] where the most marked changes are in the levels of synaptophysin and VAMP-1 [29]. Treatment of pregnant dams in the present study did not produce any significant change in the expression of synaptophysin or the synaptic vesicle calcium sensor protein synaptotagmin. This result is consistent with a study by Afadlal et al. [31] in which prenatal stress induced by maternal restraint or corticosterone injections produced a decrease in synaptophysin levels only in animals at P7 or P14, but not at later ages such as the P21, post-weaning time examined here. On the other hand the levels of VAMP-1 were affected to a significant degree, emphasising the potential parallels between the effects of poly(I:C) and the changes reported in schizophrenia [29].

An increasingly recognized view of the molecular changes underlying schizophrenia postulates a hypofunction of NMDA receptors. It is already established that neuronal receptors for glutamate, especially those sensitive to NMDA, affect neuronal migration [32], synapse formation [33], and neurite growth [34], spine formation [35,36] and neuronal plasticity [6,37,38]. The pharmacological blockade of NMDARs in neonatal rats causes a loss and disruption of synapses with profound abnormalities of brain structure and behaviour in adulthood [39-41], many of which resemble the behavioural abnormalities which occur in schizophrenia [42]. Thus, an induced change in NMDA receptor function during development could modify (for example by accelerating or slowing, amplifying or suppressing) the normal processes of programmed neuronal elimination which shapes the early nervous system.

In light of this fundamental role of NMDAR in neuron and synapse formation and function, the present finding of substantially reduced expression of the major subunit of these receptors could imply that a change in NMDAR number or function could underlie some of the neurochemical and behavioural abnormalities which have been described after exposure of animals to LPS or poly(I:C) [21,23,24]. NMDARs are composed of GluN1 and GluN2 subunits which bind glutamate and exert a regulatory control over GluN1. The GluN1 subunit is encoded by one gene to produce several splice variants. The antibody used in this work was selected as it interacts with all of the known splice variants. The change in GluN1 expression, therefore, reflects a real overall change of NMDAR number or function. Changes similar to those observed here have been reported previously in the expression of GluN1 receptors, although the experimental design was substantially different, with LPS injections or viral infection being induced directly in the pups themselves during postnatal development, and not during gestation [40,43]. Nevertheless, taken together, these various studies offer highly suggestive mutual support for the concept that activation of the immune system can interfere with NMDAR function during brain development.

The small GTPases RhoA and RhoB have repeatedly been linked with synaptic plasticity [16,17,44] and various aspects of neuronal development [45,46], often mediating the effects of NMDAR. However, there were no clearly significant changes in their expression. Similarly, despite the role of the tyrosine kinase family of Eph receptors and their ephrin ligands in synaptogenesis [47] and spine formation [48-50] the absence of any clear change in the level of EphA4 implies that it does not contribute to changes in neuronal development and behaviour produced by poly(I:C).

There was a significant increase in the expression of Sox-2 (sex-determining region Y-related HMG-box2), a transcription factor involved in stem cell maintenance in the brain as well as a major factor in regulating cell loss or survival during development [51]. Sox-2 is often considered a marker of cells that are actively dividing but which are in the early phases of proliferation and are as yet undifferentiated [52]. Sox-2 occurs primarily in brain regions that contain actively proliferating neural stem cells, possibly maintaining a ‘latent’ and undifferentiated state [53,54]. Suppression or deletion of sox-2 can lead to increased apoptosis [19] and is incompatible with embryonic survival [55], although overexpression does not promote proliferation [56]. The pattern of changes seen in this group of neurogenesis markers, therefore, suggests a possible increase in the number of cells (as yet undifferentiated) at an early stage of proliferation. However, since no change was detected in the expression of PCNA, a nuclear protein which is increasingly used as a marker for the early phases of cell degeneration [51], the sox-2 expressing cells either do not express this protein at P21, or they have past the stage of producing PCNA. Similarly there was no change in levels of doublecortin, a molecule associated primarily with later stages of neurogenesis and whose expression declines during brain development. This observation suggests that poly(I:C) administration does not produce significant overall changes in neuronal or glial generation at P21.

A major question which arises following the induction of inflammation by poly(I:C) or bacterial polysaccharides (LPS) is whether any observed effects are a direct action of the injected agent, or are secondary, indirect consequences mediated via an intermediate compound. It is improbable that the immunostimulator molecules are themselves responsible, since they are unlikely to cross into embryos. In one study, radiolabelled LPS was administered to pregnant dams but the labelled compound was detected only in maternal tissues and not in the embryos [57].

The compounds that have been most often considered in the role of mediating immunostimulant effects in the brain are the cytokines. It is well recognised that both poly(I:C) and LPS can induce the expression and release of several pro-inflammatory cytokines although they can also modify the expression of neurotrophic proteins such as brain-derived growth factor (BDNF) and nerve growth factor (NGF), all of which have profound effects themselves on the CNS.

The administration of poly(I:C) does increase plasma levels of IL-1β, IL-6, TNF-α and interferon-β in adult rats when measured at the appropriate time points [12,13] and both IL-1β and TNF-α have been detected within the brain after systemic administration of LPS or poly(I:C) [58-60]. This expression of cytokines has also been shown after maternal administration of LPS, when expression of IL-1β, IL-6 and TNF-α, as well as MCP-1 was demonstrated within the brains of the embryos [61]. These results seem to depend acutely on the precise experimental protocol since others have found that the expression of TNF-α was reduced by LPS in the foetal brain, whereas poly(I:C) had no effect [12]. Other workers have reported finding either no change of foetal brain TNF-α [62] or an increase [63].

Some of the most compelling work is that which shows the ability of cytokine antagonists to prevent CNS features of inflammation such as microglial activation, neuronal death or cognitive dysfunction. Some of these effects can be prevented by an IL-1β receptor antagonist [58,64], although the direct administration of IL-1β to pregnant dams has been reported not to result in changes in the offspring.

Certainly, both neurons and glial cells possess interleukin receptors and there have been many studies showing that a range of cytokines, including IL-1β, have marked effects in the CNS even after peripheral delivery [65]. Work by Riazi et al., [59,60] has shown that poly(I:C) can increase the CNS levels of IL-1β, the cytokine which was probably responsible for the observed increase in CNS excitability and susceptibility to seizures induced by pilocarpine or pentylenetetrazol. Interestingly these effects were accompanied by an increased expression of GluN2A and GluN2C subunits which did not occur in the present study, presumably because of the difference between direct administration into the postnatal brain rather than administration into the mother followed by examination of the offspring.

Although most attention on cytokine mediation of poly(I:C) effects has been concentrated on IL-1β, there are several alternative candidates, such as IL-6. When labelled IL-6 was injected into pregnant rats, the protein was found in the amniotic fluid and in the foetus itself [66] showing its ability to pass across the placental barrier. In addition, maternal IL-6 administration has been reported to reproduce many of the effects of poly(I:C) on offspring, a claim strongly supported by the finding that antagonism of IL-6 prevented the effects of poly(I:C) [9,10].

The problem of identifying a likely mediator of poly(I:C) effects is complicated further by the fact that neurons and glia can express several cytokines including IL-1β and TNF-α [67], so that the passage of cytokines into the CNS may not even be required for them to mediate genetic changes within the brain. It may only be necessary that neurons or glia are activated by a small molecule factor which can pass readily between blood and brain. However, Oskvig et al. [68] have noted that cytokine protein levels are increased within the foetal brain whereas the corresponding RNA molecules are not, implying that the foetal proteins are derived from the maternal circulation and not from foetal synthesis. There is evidence that poly(I:C) can increase the permeability of barriers [69] and it may therefore contribute indirectly to an increased movement of cytokines into the foetus.

Overall, the profile of molecular changes described here suggests that, after exposure in utero to the viral mimetic poly(I:C), rat pups exhibit abnormal levels of several proteins known to be important in cerebral development. Even though several of the proteins studied show no significant change, it is important to realise that the absence of a change may be as scientifically important as the presence of a change. Firstly, it is valuable evidence that the schedule of immune activation used in the study does not produce a non-selective toxicity, with a generalised loss of cells and proteins. Secondly, when development of the brain can be understood in terms of the full gamut of molecular changes of expression it is likely to be just as important to know which aspects of neuronal development are unaffected by inflammation as to know which are. In addition, it is highly likely that for many aspects of brain development it is the relative change between molecules that may be the determining factor in abnormality, especially if different molecular changes occur at different rates during the course of development. Here again therefore it will be essential to know which proteins change in response to insult and which do not.

Finally, we elected to examine the overall protein expression in cerebral hemispheres. The apparent absence of a change may, therefore, mask more localised, significant changes, which need to be investigated in more detail.

## Conclusions

By 21 days of age, following the prenatal exposure of rats to the viral mimetic poly(I:C), there is clear evidence for changes in the expression of one of the major proteins associated with neurotransmitter release (VAMP-1), one involved in NMDA receptor signaling (GluN1) and one implicated in early neuronal development (sox-2). In view of the various roles of NMDA receptors in neuronal development, these changes could contribute to an altered rate of new cell production and differentiation reflected in the altered sox-2 expression. These changes not only lend strong support to the concept that activation of the immune system during gestation can lead to abnormal brain development, but also emphasise the central importance of NMDA receptors. The results are of particular importance to disorders such as schizophrenia, in which reduced activation of NMDA receptors appears to be a major cause or trigger of the behavioural abnormalities, and for which therapies designed to enhance or facilitate NMDA receptor function may be of clinical value.

## Abbreviations

GluN1, GluN2A, GluN2B: Glutamate receptor subunits; HRP: Horseradish peroxidase; IFN-γ: Interferon-γ; IL-1β: Interleukin-1β; IL-6: Interleukin-6; LPS: Lipopolysaccharides; NMDA: N-methyl-D-aspartate; MCP-1: Monocyte chemoattractant protein-1; PCNA: Proliferating Cell Nuclear Antigen; poly(I:C): poly-[inosinic acid:cytidylic acid]; PSD-95: Post-Synaptic Density molecule-95; RhoA,RhoB: Members of the Ras (rat sarcoma) family of small GTPase enzymes; Sox-2: Sex-determining region Y-related HMG-box2; TBST: Tris-buffered saline containing 0.05% Tween; TLR-3: Toll-like receptor-3; TNF-α: Tumour necrosis factor-α; VAMP-1: Vesicle Associated Membrane Protein-1.

## Competing interests

The authors declare that they have no competing interests.

## Author contributions

CMF established the experimental protocol, CMF, OSK and MP acquired the experimental data and performed the analysis; TWS conceived and directed the study and drafted the initial manuscript; LGD and RAS contributed to development of the protocols, direction of the project, interpretation of data and modifying the draft manuscript. All authors have reviewed and approved the final manuscript.
